# The Effect of Silver Nitrate Pleurodesis after a Failed Thoracoscopic Talc Poudrage

**DOI:** 10.1155/2013/295890

**Published:** 2013-09-01

**Authors:** Cecilia Menna, Claudio Andreetti, Mohsen Ibrahim, Giulio Maurizi, Camilla Poggi, Rocco Barile, Francesco Cassiano, Erino A. Rendina

**Affiliations:** ^1^Division of Thoracic Surgery, “G. Mazzini” Hospital of Teramo, Faculty of Medicine and Surgery, University of L'Aquila, Piazza Italia 1, 64100 Teramo, Italy; ^2^Division of Thoracic Surgery, Sant'Andrea Hospital, Faculty of Medicine and Psychology, University of Rome “Sapienza”, Via di Grottarossa 1035, 00189 Rome, Italy; ^3^Fondazione Eleonora Lorillard Spencer Cenci, Via Di Casal de' Pazzi 10, 00156 Rome, Italy

## Abstract

*Background*. Chemical pleurodesis is the procedure of choice in the management of recurrent malignant pleural effusions (MPE). Talc is probably the most effective sclerosant, with a success rate of 80%. The aim of this study is to demonstrate the effectiveness of silver nitrate solution (SNS) pleurodesis after an unsuccessful thoracoscopic talc poudrage. *Methods*. Between 2011 and 2013 one hundred and nine patients with unilateral MPE underwent thoracoscopic talc poudrage. Seventeen patients who did not obtain a successful pleurodesis via thoracoscopic procedure were considered for an SNS slurry. The pleural injectate consisted of 100 mL 1% SNS with 10 mL of lidocaine (100 mg/5 mL). The SNS procedure was undertaken once and repeated with the same dose in 5 patients. *Results*. The duration of follow-up period was 30 days. Subjective pain was low and the same before and after SNS procedure (*P* value = NS). The mean daily fluid drainage was statistically different (*P* = 0.001) comparing values before (597.0 ± 122.8 mL) and after SNS procedure (109.1 ± 22.3 mL). After 30 days from SNS procedure recurrence of pleural effusion was observed in 2 patients (11%). *Conclusions*. The present study demonstrates that SNS is an effective agent for producing pleurodesis after a failed thoracoscopic talc poudrage.

## 1. Introduction

Chemical pleurodesis using various sclerosing agents is accepted as a palliative therapy for patients with recurrent, symptomatic, and malignant pleural effusions (MPE) [1]. The ideal sclerosing agent should be effective, cheap, readily available, easy to handle, and well tolerated with no significant side effects. Talc is probably the most effective sclerosant, with a success rate of 80% [2]. Silver nitrate solution (SNS) is a valid alternative sclerosant, inducing a caustic injury to the mesothelium that results in an effective pleurodesis. The efficacy and the tolerability of SNS treatment in an animal model of pleural effusion and in human have been recently demonstrated [3, 4]. However, various clinical parameters and biochemical factors affect the success of pleurodesis in symptomatic patients with MPE: symptoms and performance status of the patient, daily fluid drainage, primary tumour, and mainly lung reexpansion following pleural fluid evacuation [2, 5, 6]. Longer time period between the diagnosis of MPE and onset of treatment could increase recurrence [7]. Concerns predicting pleurodesis outcome are still controversial [8–10]. According to the BTS Pleural Disease Guidelines 2010, chemical pleurodesis may still be attempted in patients with large pleural effusions or “trapped lung” where pleural apposition cannot be achieved, after a previous failed pleurodesis. Other effective options are described for large pleural effusion refractory to chemical pleurodesis, such as chronic indwelling pleural catheter drainage or pleuroperitoneal shunts [11–13]. To avoid alternative more invasive procedures, we aim to demonstrate the effectiveness of SNS pleurodesis after an unsuccessful thoracoscopic talc poudrage.

## 2. Materials and Methods

### 2.1. Patients

Between January 2011 and March 2013, one hundred and nine patients with unilateral MPE underwent thoracoscopic talc poudrage at the Division of Thoracic Surgery, Sant'Andrea Hospital, Rome. All patients had a documented MPE, a Karnofsky index score of >60, a life expectancy >1 month, and no preoperative evidence of loculated or trapped lungs after drainage. Surgery was performed by the same equipe under general anesthesia with selective bronchial intubation. Patients were placed in lateral decubitus with the arm abducted at 90°. A single-port access was performed at the seventh or eighth intercostal space inserting o°, 5 mm camera and endoscopic dissector or the catheter to insufflate 4 g of micronized sterile talc powder (Steritalc, Novatech SA, La Ciotat, Cedex, France). Antibiotic prophylaxis (i.v. sodic cefazolin, 1 g × 2/day) was administrated in all patients until chest tube removal. Seventeen patients who did not obtain a successful pleurodesis via thoracoscopic procedure were considered for an SNS slurry. An unsuccessful pleurodesis was defined by the evidence of more than 300 mL as a daily drainage volume after 7 days from the thoracoscopic talc poudrage. The study was approved by the Ethics Committee of our Institution, and a written informed consent from all patients was obtained. Hospital stay, chest tube permanence, and side effects (fever, ARDS, and empyema) were evaluated after SNS slurry. The mean daily fluid drainage was assessed before and after SNS procedure. Outcomes at 3 and 6 months after SNS procedure were reported.

### 2.2. SNS Slurry Technique and Pleurodesis Assessment

All patients had a chest tube (28 Ch) inserted at the end of the thoracoscopic procedure. After the 7th day of large pleural effusion persistence (>300 mL), SNS was then injected through the chest tube. The pleural injectate consisted of 100 mL 1% SNS (galenic formulation from the Pharmacy of our Institution) with 10 mL of lidocaine (100 mg/5 mL). The sclerosant was injected, and the chest tube was clamped for 20 minutes, placing the patient in the prone, supine, right and left decubitus positions during this time. The chest tube was then unclamped. No suction was applied after SNS slurry, and drainage was left watersealed. Serial radiographs were used to document appropriate lung reexpansion. The first was performed 24 h after the intrapleural injection. Then, after patients' discharging, chest X-Ray was performed at 14 and 30 days when they come back to visit in their follow-up. Patients were followed up every day and discharged when the amount of fluid collected in the previous 24 h was <150 mL, with the chest tube being removed. The SNS slurry was undertaken once, with the procedure with the same dose in 5 patients being repeated. In these cases the procedure was repeated after four days from the first SNS slurry. All patients were clinically evaluated before and 1 h after treatment regarding pain score using a VAS scale from 0 to 10 (0 = no pain, 10 = the worst pain). Each patient was asked to provide a number describing his/her pain at the moment. Patients were requested to return 30 days after SNS pleurodesis with a chest radiograph. At this visit, patients were considered to have a successful pleurodesis if there was no recurrence of the pleural effusion.

### 2.3. Statistical Analysis

Data are expressed as mean ± SE and median. Student's unpaired *t*-test was used to compare continuous variables before and after SNS treatment.

## 3. Results and Discussion

### 3.1. Results

Seventeen patients received an intrapleural injection with SNS. Ninety-two patients were not considered for the study because they received a successful thoracoscopic talc pleurodesis within 7 days from the procedure. The characteristics of patients are shown in Table 1. The duration of follow-up period was 30 days to assess the effectiveness of SNS procedure. Further outcomes at 3 and 6 months are reported, except for 1 patient who did not reach a 6-month-follow-up period (undergoing surgery in March 2013).

**Figure 1:**
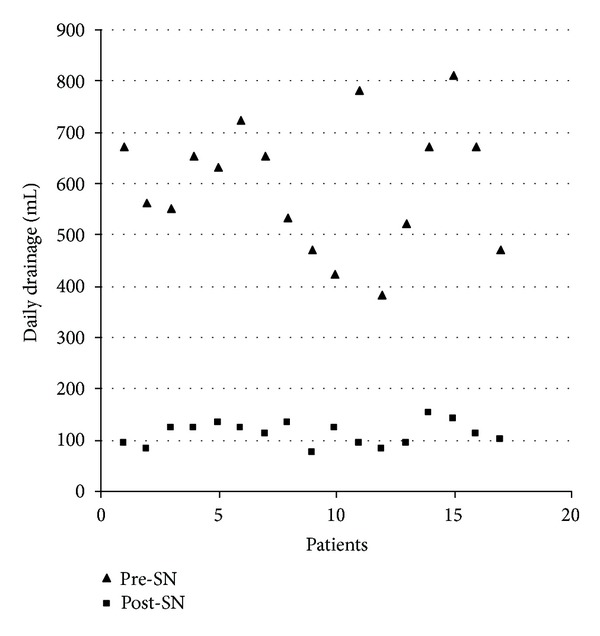
Comparison between daily fluid drainage (mL) before and after silver nitrate (SN) procedure (*P* < 0,001).

**Figure 2:**
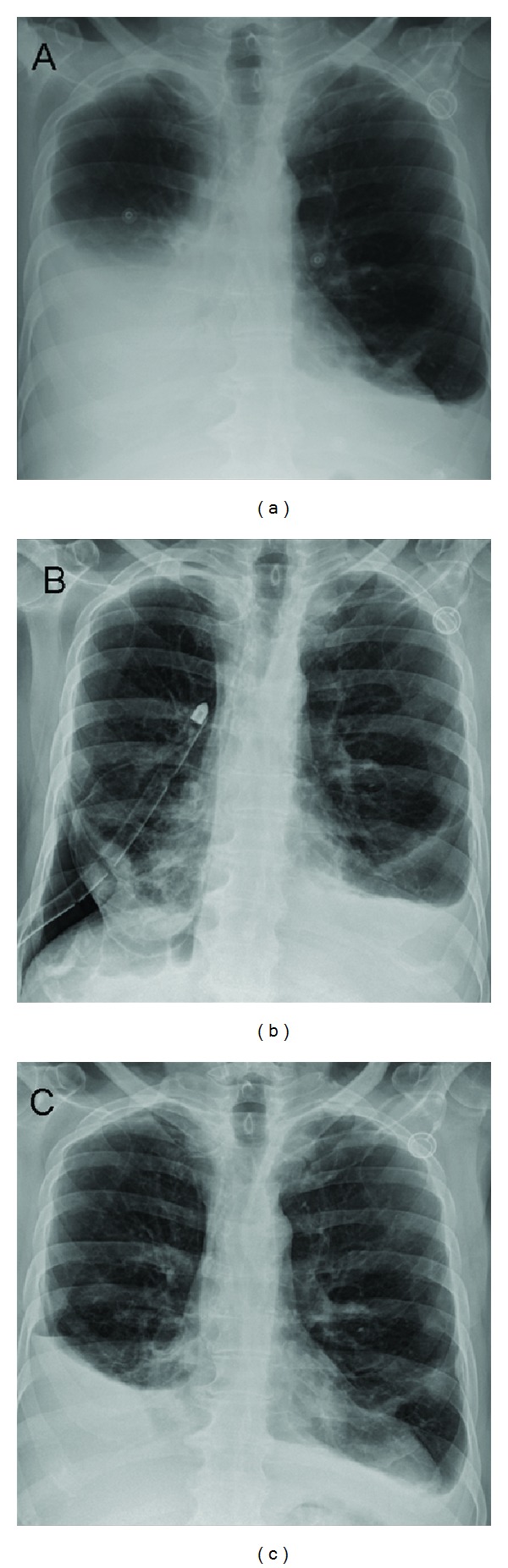
(a) Preoperative chest X-Ray, (b) chest X-Ray one day after thoracoscopic talc poudrage in a patient who received an unsuccessful pleurodesis, and (c) chest X-Ray 30 days after SNS slurry. (Images a, b, and c are from the same patient who received a repeated administration of SNS.)

SNS was found to be effective as a pleurodesis agent in the present study, and the results are shown in Table 2. Subjective pain was low and the same before and after SNS procedure (3.4 ± 1.3 versus 3.7 ± 1.6, resp., *P* value statistically not significant). The mean daily fluid drainage was statistically different (*P* = 0.001, Figure 1) comparing the values before (597.0 ± 122.8 mL) and after SNS procedure (109.1 ± 22.3 mL). The mean number of days spent in the hospital after SNS slurry was 8.2 ± 2.8. The mean chest tube permanence after SNS slurry was 6.2 ± 2.3, and all patients were discharged without the chest tube. All patients intraoperatively showed a large macroscopic involvement of parietal and/or visceral pleura(s) (>90%) and a severe haematic effusion. Nevertheless, in five patients (30%) a poor intraoperative lung reexpansion and a daily drainage volume >250 mL after SNS slurry were observed, with a repeated administration of SNS being repeated (at the same dose) after four days from the first slurry (Figures 2(a)-2(b)). However, recurrence of pleural effusion was observed in 2 patients (11%) 30 days from the SNS slurry (Figure 2(c)). Moreover, five patients (29%) developed a transient elevation of temperature (>37°C) the day after the SNS slurry, while four patients (23%) developed the same side effect after the talc poudrage taking into consideration the same group of patients (*P* value statistically not significant). None of the patients experienced ARDS or pleural empyema after SNS procedure. None of the patients who returned for followup had other significant side effects from the SNS agent (i.e., coughing, chest tightness, and reexpansion edema). No mortality was registered because of the SNS procedure and no patients died during the 30-day follow-up period. No other adverse events were reported at 3 and 6 months after SNS procedure. Moreover, 100% of patients died within 6 months after SNS procedure because of the extended disease (82% of patients, *n* = 14, died within 3 months).

### 3.2. Discussion

Treatment options for MPE are determined by several factors: symptoms and performance status of the patient, the primary tumor and its response to systemic therapy, and lung reexpansion following pleural fluid evacuation. Observation is indicated for small and asymptomatic effusions, while therapeutic thoracentesis is recommended for the palliation of breathlessness. However, thoracentesis is characterized by the high recurrence rate and the risk of pneumothorax. Chest tube insertion with intrapleural sclerosant slurry could reach a success rate of more than 60%. However, thoracoscopy with talc poudrage ensures the highest success rate (80%), albeit it is considered an invasive procedure and it may be unavailable in some hospitals.

Although chemical pleurodesis is the procedure of choice in the management of recurrent MPE, the most important requirement for successfull pleurodesis is satisfactory apposition of the parietal and visceral pleuras, confirmed radiologically. Incomplete lung reexpansion may be due to a thick visceral peel (“trapped lung”), pleural loculations, proximal large airway obstruction, or a persistent air leak. Most studies indicate that the lack of a response following instillation of a sclerosant is predominantly due to incomplete lung expansion [14, 15]. If complete lung reexpansion or pleural apposition is not achieved and the patient is unsuitable for surgical intervention, pleurodesis should be still attempted. The amount of pleural fluid drained per day before the instillation of the sclerosant (<150 mL/day) is less relevant for successfull pleurodesis than radiographic confirmation of fluid evacuation and lung reexpansion. Randomized studies have shown a better outcome in patients who received a sclerotherapy as soon as complete lung reexpansion was documented (<24 h) than in patients who received pleurodesis only when the fluid drainage was <150 mL/day [14]. It has been demonstrated that, although a strong activation of coagulation and production of PAI (plasminogen activator inhibitor) was observed in patients with pleural effusion, increased pleural fibrinolytic activity is associated with failure of pleurodesis [16].

Once a patient is considered to be appropriate for pleurodesis, a sclerosing agent must be chosen. The ideal sclerosant should produce, in the shortest possible time, an effective and safe obliteration of the pleural space. Moreover, it should be inexpensive and readily available and require a short hospital stay. To date, talc, doxycycline, iodopovidone, and bleomycin are the sclerosing agents commonly used. Although molecular antineoplastic sclerosing agents (i.e., anti-VEGF, anti-MMPs, and anti-COX2) have been studied for the treatment of MPE [17], to date, no selective biological agent of choice exists. Identification of new target sclerosing therapies is hampered by the lack of comparative randomized trials and different eligibility criteria.

On the other hand, the management of that 20% of patients who received a failed talc pleurodesis still remains an open point since strong guidelines are not present. To date, little investigations have been conducted to overcome this issue.

In the present study, we show the efficacy of single or repeated intrapleural administration of low concentrations of SNS in controlling recurrent MPE after thoracoscopic talc poudrage.

The intrapleural injection of SNS, with the objective of producing effective pleurodesis, was proposed in the 1940s. It was first used in 1942 by Brock [18] to produce aseptic pleural adhesions. In 1948, the same author proposed the intrapleural instillation of 10% SNS for the treatment of spontaneous pneumothorax [19–22]. Since then, SNS has been used sporadically, probably due to severe pain occurring after the procedure [21].

Lower concentrations may also be effective. Stowe and coworkers [23] instilled a 1% SNS solution in 10 patients and reported only one recurrence. In contrast, Schuster and coworkers [24] injected a 1% SNS solution into the pleural space of eight patients and reported that three patients had a recurrence.

The degree of injury is important in determining whether a pleurodesis will result, and it is important to produce the correct degree of injury. Intrapleural injection of a 10% SNS solution injures the pleura too severely, resulting in severe pain and large pleural effusions. However, it has been demonstrated in humans that lower concentrations of SNS might produce a degree of pleural injury sufficient to induce pleurodesis without the severe pain and large effusions [25, 26]. This hypothesis was confirmed in rabbits [27, 28].

Moreover, SNS can be considered a valid therapeutic option in selected patients presenting with an early bronchopleural fistula as reported from Andreetti et al. [29].

In our study, single or repeated dose of low concentration of SNS (1%) produced an effective pleurodesis in 89% of patients after a failed thoracoscopic talc poudrage during the first 30 days after treatment, with moderate side effects. Only 29% of patients presented with fever after SNS slurry, and none of the patients showed ARDS occurrence. No significant problems with pain were observed in any of the patients. The medium peak pain score obtained during the hospitalization period after SNS slurry was 3.7 ± 1.6. Although no other significant side effects either in the short term or the long term were observed in this study, we cannot draw unequivocal conclusions regarding the safety of SNS since the number of patients receiving SNS was small, and the overall follow-up period was relatively short. However, the extended disease demonstrated by the presence of MPE itself could not allow having a larger sample of patients and a longer follow-up period. Patients eligible for SNS slurry belong to that 20% of patients who received a failed talc poudrage. A further shortcoming of the present study is the lack of a comparison with a control treatment group that could be overcome with a randomized clinical trial and blinded investigators.

## 4. Conclusion

In conclusion, the present study demonstrates that SNS is an effective agent for producing pleurodesis after a failed thoracoscopic talc poudrage. Moreover, the side effects of intrapleural SNS at a concentration of 1% appear to be minimal. Thus, SNS should be considered as a viable alternative to be introduced into the clinical practice.

## Figures and Tables

**Table 1:** Clinical characteristics of patients.

Variables	*n*	%
Sex (male/female)	11/6	64/36
Age (mean ± SD, years)	62.4 ± 21.3	—
Karnofsky index		
≤80	41	82
>80	9	18
Side (left/right)	7/10	41/59
Pathology		
Lung	5	30
Breast	4	23
Kidney	3	17
Others	5	30

SD: standard deviation.

**Table 2:** Results.

Variables	Before SNS slurry	After SNS slurry	*P* value
Pain score (mean ± SD)*	3.4 ± 1.3	3.7 ± 1.6	NS
Fluid drainage (mean ± SD)^†^	597.0 ± 122.8	109.1 ± 22.3	0.001
Hospital stay (mean ± SD)	7 ± 0	8.2 ± 2.8	NS
Chest tube permanence (mean ± SD)	—	6.2 ± 2.3	—
Recurrence in 30 days (*n*/%)	—	2/11	—
Fever (*n*/%)	4/23	5/29	NS
ARDS (*n*/%)	—	0/0	—
Empyema (*n*/%)	—	0/0	—
Mortality (*n*/%)	—	0/0	—

SD: standard deviation.

*Pain was evaluated before and 1 h after the first administration of SNS.

^†^Considering the mean daily fluid drainage during the postprocedure period, both after talc poudrage and SNS slurry.
